# Role of Circulating Angiotensin Converting Enzyme 2 in Left Ventricular Remodeling following Myocardial Infarction: A Prospective Controlled Study

**DOI:** 10.1371/journal.pone.0061695

**Published:** 2013-04-22

**Authors:** José T. Ortiz-Pérez, Marta Riera, Xavier Bosch, Teresa M. De Caralt, Rosario J. Perea, Julio Pascual, María José Soler

**Affiliations:** 1 Thorax Institute, Hospital Clínic, Institut d’investigacions Biomèdiques August Pi i Sunyer, University of Barcelona, Barcelona, Spain; 2 Nephrology Department, Hospital del Mar, Institut Hospital del Mar d’investigacions Mèdiques, Barcelona, Spain; 3 Center for Diagnostic Imaging, Radiology Department, Hospital Clínic, University of Barcelona, Barcelona, Spain; Universityhospital Düsseldorf, Germany

## Abstract

Angiotensin-converting enzyme 2 (ACE2) cleaves Angiotensin-II to Angiotensin-(1–7), a cardioprotective peptide. Serum soluble ACE2 (sACE2) activity is raised in chronic heart failure, suggesting a compensatory role in left ventricular dysfunction**.** Our aim was to study the relationship between sACE2 activity, infarct size, left ventricular systolic function and remodeling following ST-elevation myocardial infarction (STEMI). A contrast-enhanced cardiac magnetic resonance study was performed acutely in 95 patients with first STEMI and repeated at 6 months to measure LV end-diastolic volume index, ejection fraction and infarct size. Baseline sACE2 activities, measured by fluorescent enzymatic assay 24 to 48 hours and at 7 days from admission, were compared to that obtained in 22 matched controls. Patients showed higher sACE2 at baseline than controls (104.4 [87.4–134.8] vs 74.9 [62.8–87.5] RFU/µl/hr, p<0.001). At seven days, sACE2 activity significantly increased from baseline (115.5 [92.9–168.6] RFU/µl/hr, p<0.01). An inverse correlation between sACE2 activity with acute and follow-up ejection fraction was observed (r = −0.519, p<0.001; r = −0.453, p = 0.001, respectively). Additionally, sACE2 directly correlated with infarct size (r = 0.373, p<0.001). Both, infarct size (β = −0.470 [95%CI:−0.691:−0.248], p<0.001) and sACE2 at 7 days (β = −0.025 [95%CI:−0.048:−0.002], p = 0.030) were independent predictors of follow-up ejection fraction. Patients with sACE2 in the upper tertile had a 4.4 fold increase in the incidence of adverse left ventricular remodeling (95% confidence interval: 1.3 to 15.2, p = 0.027). In conclusion, serum sACE2 activity rises in relation to infarct size, left ventricular systolic dysfunction and is associated with the occurrence of left ventricular remodeling.

## Introduction

The activation of the renin-angiotensin-aldosterone system (RAAS) is a well-known final pathway following ST-elevation-myocardial infarction (STEMI), leading to adverse left ventricular (LV) remodeling, heart failure and cardiac death. In addition to the cardioprotective effects provided by beta-blockers, it is well established that pharmacological blockade of the RAAS with Angiotensin-converting enzyme (ACE) inhibitors, Angiotensin-II receptor blockers or aldosterone antagonists limit LV remodeling and improve prognosis following STEMI [1], [2]. However, despite optimal medical treatment with these drugs, many STEMI patients develop adverse LV remodeling or heart failure during follow-up [3].

Angiotensin-converting enzyme 2 (ACE2) is an analogue of the ACE that cleaves Angiotensin-II into Angiotensin-(1–7), a peptide with vasodilatory properties including an increase in coronary perfusion and attenuation of post-ischemic LV dysfunction that antagonizes angiotensin-II actions [4]. ACE2 deficiency in mice increases angiotensin-II, which causes severe LV dilatation and systolic dysfunction that is reversed by genetic deletion of ACE [5]. On the other hand, administration of recombinant human ACE2 attenuates angiotensin-II and pressure-overload induced adverse LV remodeling, suggesting that ACE2 is an important negative regulator of angiotensin-II induced heart disease [6].

Recently, it has become feasible to measure soluble ACE2 (sACE2) activity in human serum, which allows the non-invasive study of this component of the RAAS. Serum sACE2 activity has been also shown to correlate with the presence and severity of heart failure among patients with ischemic and non-ischemic cardiomyopathy, to reinforce a cardioprotective and compensatory role in humans [7]. Therefore, ACE2 may potentially exert beneficial biological effects following STEMI as opposed to ACE [8], [9]. We hypothesized that sACE2 activity would be increased in STEMI patients and would correlate with infarct size and the extent of LV dysfunction as assessed by contrast enhanced cardiac magnetic resonance imaging (ce-CMR).

## Methods

### Ethics Statement

Both, the Hospital Clinic of Barcelona Research Committee and the Ethics Committee for Clinical Research approved this study. All participants and control subjects signed a consent form.

### Patient Population and Sample Collection

**Figure 1:**
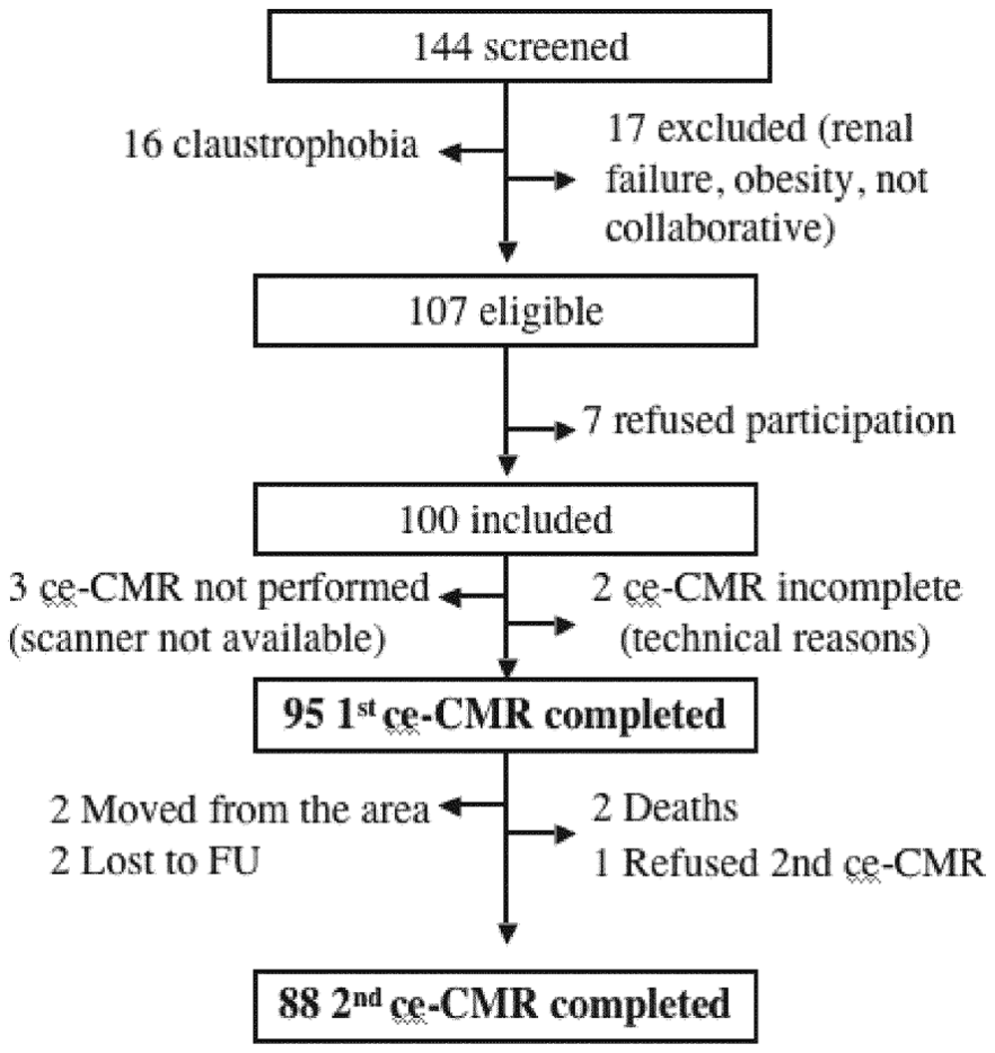
Patient selection. Flow-chart detailing patient selection. ce-CMR: contrast-enhanced Cardiac Magnetic Resonance; FU: follow-up.

From January 15^th^, 2009 to January 31^st^, 2010, 270 patients without prior history of cardiac disease were admitted to the Coronary Care Unit following STEMI. A total of 98 stable patients were immediately transferred to the referring hospital following reperfusion and were not assessed for this study. There were 8 early deaths and other 20 patients with clinical instability were excluded. In all, 144 patients were screened for their participation in the study. Further patient selection is detailed in Figure 1. Ninety-five patients who completed the first ce-CMR formed the study group. Of them, 88 (93%) returned for the follow-up ce-CMR. The standard of care in treating STEMI was applied. Primary percutaneous intervention was the reperfusion treatment, delivered by experienced on-call interventional cardiologists following unfractionated heparin, aspirin and a loading dose of clopidogrel. At the physician’s discretion and unless contraindicated, captopril or enalapril (at least 6.25 mg every 8 hours or 2.5 mg every 12 hours, respectively), and beta-blockers were initiated early, usually by 24 hours from admission. Serum troponin I was measured during 48 hours, every 6 hours during the first 12 hours and every 12 hours thereafter. In addition, serum B-type natriuretic peptide (BNP) measured 48 hours after admission, was available in 76 cases. To determine sACE2 activity at baseline, blood samples (10 ml) were drawn between 24 and 48 hours after symptoms onset (mean 34±5 hours), and at 7 days. A single blood sample was drawn in a control group, formed by 22 subjects without known cardiovascular disease selected from a general medicine outpatient clinic. All blood samples from patients and controls were centrifuged and post-processed similarly. The isolated serum was stored at –80°C until analysis, which was performed the same day using the same assay.

### ACE2 Enzymatic Assay

The ACE2 fluorescent enzymatic assay protocol was performed as previously described with modifications, using an ACE2 quenched fluorogenic substrate (Mca-Ala-Pro-Lys(Dnp)-OH, Enzo Life Sciences) [10], [11]. Briefly, 20 µL of serum was incubated with a buffer (100 mM Tris-HCl, 600 mM NaCl, 10 µM ZnCl_2_, pH 7.5) in the presence of protease inhibitors, including 100 µM captopril, 5 µM amastatin, 5 µM bestatin (all from Sigma-Aldrich), and 10 µM Z-Pro-prolinal (Enzo Life Sciences). Samples were incubated with 20 µM quenched fluorogenic substrate in reaction buffer (final reaction volume 100 µL) at 37°C. Specificity was determined by pre-incubating serum for 30 minutes with or without 0.6 µM DX600 (Phoenix Pharmaceuticals), a specific ACE2 inhibitor [12]. Serum sACE2 activity was determined at 18 hours. The plates were read using a fluorescence plate reader (Tecan Infinite 200, TECAN Instruments) at an excitation wavelength of 320 nm and an emission wavelength of 400 nm. Results are expressed as relative fluorescence units (RFU). To assess the intra and interobserver variability of sACE2 activity, a set of 44 serum samples was randomly selected. Then, two volumes of serum (20 µL) were analyzed in parallel for each serum sample, and all assays were repeated using the same methodology with one-week interval between them. The intraassay variability for sACE2 activity was 4.72±0.30%, and the interassay variability was 6.23±0.75%.

To explore whether sACE2 release was related to a membrane shedding effect, we measured serum levels of tumor necrosis factor-∝ converting enzyme ADAM17 using DuoSet ELISA development kit (R&D Systems, Minneapolis, MN, USA) and following manufacturer’s instructions [13]. Samples were diluted 1∶2 for the assays, which were performed blinded to patient clinical characteristics.

### Cardiac Magnetic Resonance

A standard ce-CMR study was performed in a 1.5 T clinical scanner (CV Signa, GE, Milwaukee, USA or Symphony, SIEMENS, Erlangen, Germany) equipped with cardiac-dedicated software at a mean of 6.3±3.4 (range: 3 to 10) days of admission and repeated at 6.4±1.0 month. LV function was assessed using a steady-state free-precession cine sequence prescribed in sequential 10-mm short axis slices with no gap from base to apex to achieve full LV coverage. The myocardium at risk was assessed by edema imaging using a dark-blood T2W-SE triple inversion recovery sequence in the same short-axis positions. Delayed-enhanced images were acquired 10 to 15 min after intravenous administration of gadodiamide-DTPA (Omniscan, Amersham Health, Madrid) at a dose of 0.2 mmol/Kg using a standard segmented inversion-recovery fast gradient-echo pulse sequence. The inversion time was adjusted to null normal myocardium, typically between 180 and 320 msec. Matrix size was set to 256 and the mean field of view was 360 mm, resulting in a typical voxel size of 1.4 by 1.4 by 10 mm. All CMR images were cropped and de-identified for analysis by an independent experienced observer blinded to all clinical and angiographic data. The endocardial and epicardial borders on sequential short axis cine images were manually traced to compute LV end-systolic volume index (ESVi) and end-diastolic volume index (EDVi) as well as LV ejection fraction (EF) using Argus® software (SIEMENS, Erlangen, Germany). The hyperenhanced regions on T2 W images were manually delineated in all short axis slices to compute the myocardium at risk as a percentage of the LV wall. In 32 cases with insufficient image quality or poor signal-to-noise contrast, we used the angiographic BARI score as a surrogate of area at risk as previously reported [14].

A self-customized tool based in MATLAB platform (The MathWorks, Natick, Massachusetts) was used to compute the infarct size. First, the endocardial and epicardial borders were manually traced on short-axis contrast-enhanced images. Similarly, an experienced observer roughly outlined the hyperenhanced myocardium, and those areas were post-processed with an algorithm to define the infarcted myocardium according to the half-maximum pixel intensity criteria [15]. Briefly, a histogram of the pixel intensities is created for the entire region of interest manually drawn by the observer. Then, the software identifies the maximum pixel intensity within the region of interest. The final infarct extent is automatically defined as those pixels with a signal intensity above 50% of the maximum of the initial region. The infarcted areas on sequential short-axis slices were summed and divided by the total LV wall to calculate infarct size as a percentage of the LV wall. Microvascular obstruction (MO), defined as any area of late hypo-enhancement surrounded by hyperenhancement on ce-CMR images, was also included in the infarct size. A MO extent score was computed by summing all segments with MO according to the recommended 17-segment model.

### Statistical Analysis

Normality plots and Shapiro-Wilkoxon’s test confirmed the non-normality distribution of sACE2 activity data. Accordingly, enzymatic activities were expressed as median [25^th^–75^th^ percentiles] as well as time-to-reperfusion. Other quantitative data were expressed as mean and standard deviation. Categorical data were summarized as frequencies and percentages, and differences between groups were assessed using the chi-squared test. Comparisons of sACE2 activity between groups were performed using the non-parametric Mann-Whitney U test, and differences over time within subjects were evaluated by paired analysis. Bivariate associations between ACE2 activity, infarct size and other quantitative variables were assessed by the Spearman’s correlation coefficient. One-way analysis of variance was used among sACE2 intertertile range for the various clinical parameters. A multivariate linear regression model was constructed to identify independent significant predictors of final EF, including logarithmic-transformed ACE2 activity and other significant univariate predictors. In order to assess infarct size variability, two independent observers measured 40 randomly selected studies twice. The Lin’s concordance correlation coefficient was 0.96 (95% confidence interval: 0.94–0.98) for intraobserver variability and 0.92 (95% confidence interval:0.90–0.95). SPSS version 15.0 for Windows was used, and a p value <0.05 was considered statistically significant for all tests used.

## Results

### ACE2 Activity in Controls and Patients

**Figure 2:**
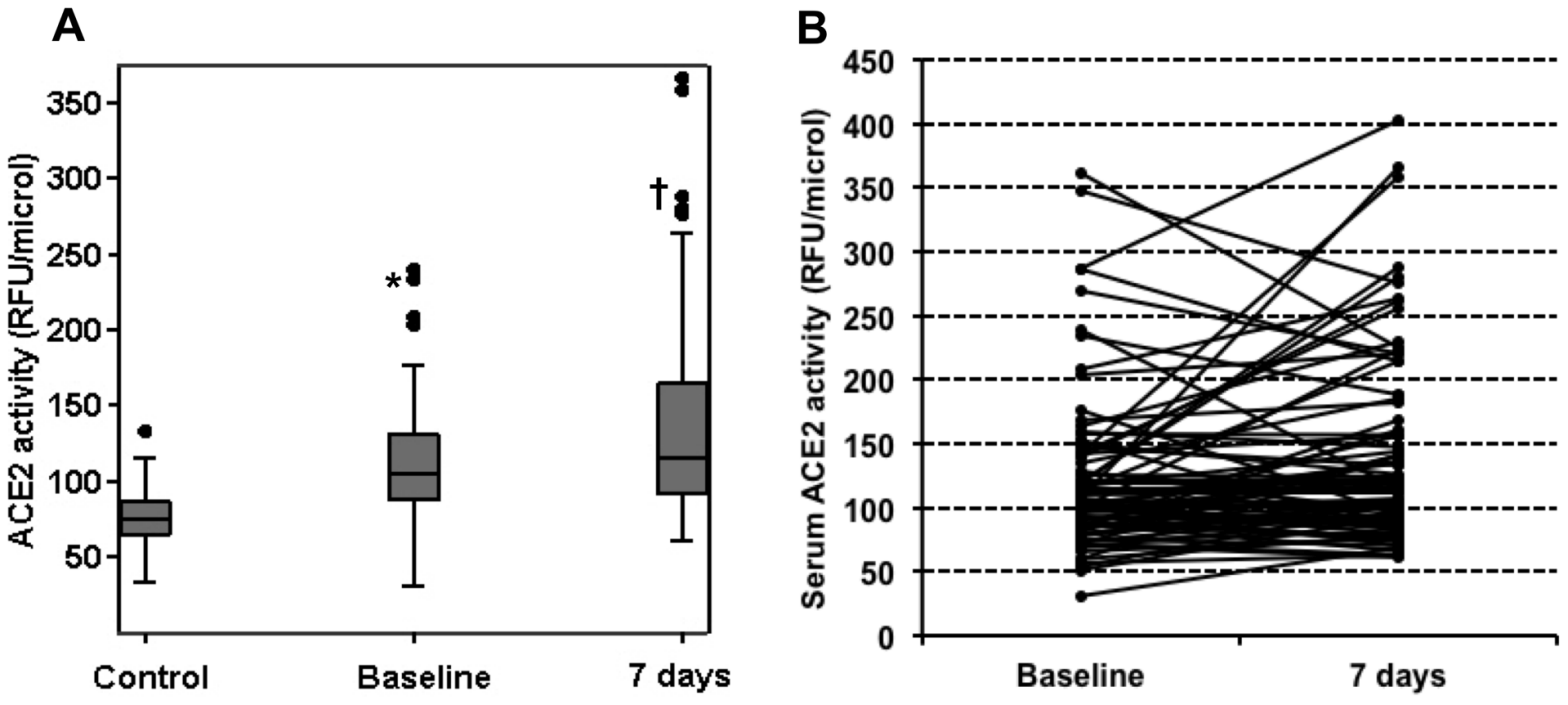
sACE2 activity in controls and patients. Panel A: Box-plot showing the differences in the median (horizontal lines), interquartile range (boxes) and 95% of the observed values (whiskers) for sACE2 activity between controls and STEMI patients. Panel B: Scatter-plot detailing individual changes in sACE2 activity among patients. *: p<0.001 for comparison between baseline measurements in patients and controls. †: p<0.01 for comparison between baseline and 7 days measurements by paired analysis. ACE2: Angiotensin Converting Enzyme 2; RFU: relative fluorometric units.

Patients (n = 95) and controls (n = 22) were well matched for sex, age, and risk factors (Table 1). The clinical status, the angiographic findings as well as the medical treatment for STEMI patients are shown in table 2. Patients showed increased sACE2 activity at baseline as compared to controls (p<0.001). Among STEMI patients, a significant increase in sACE2 activity from baseline to 7 days was observed (Table 3 and Figure 2). We found no significant association between sACE2 activity and age, diabetes, hypercholesterolemia, hypertension or tobacco use. At baseline, sACE2 activity was not significantly different among 66 subjects being treated with ACE inhibitors as compared to subjects who were not (104.9 [87.4–139.1] vs 102.6 [86.6–119.3] RFU/µl/hr, p = 0.686). No differences were also found in sACE2 activity at 7 days among 87 subjects being treated with ACE inhibitors as compared to 8 subjects who were not taking ACE inhibitors (115.3 [93.0–159.4] vs 120.8 [93.1–224.2] RFU/µl/hr, p = 0.471).

**Table 1:** Population demographic characteristics.

	STEMI patients	Controls	p value
Clinical characteristics	(n = 95)	(n = 22)	
Age (years)	58.3 (12.2)	58.9 (10.8)	0.80
Sex (% male)	82 (86.3)	16 (72.7)	0.19
Body surface area (m^2^)	1.91 (0.17)	1.91 (0.19)	0.85
Hypertension, n (%)	38 (40.0)	10 (45.5)	0.64
Diabetes, n (%)	20 (21.1)	7 (31.8)	0.27
Hypercholesterolemia, n (%)	43 (45.3)	15 (68.2)	0.06
Smokers, n (%)	72 (75.8)	13 (59.1)	0.12

Data expressed as mean (SD) or number (%).

**Table 2:** Patient characteristics.

Clinical characteristics	(n = 95)
Killip class I, n (%)	76 (80.0)
Killip class II, n (%)	17 (17.9)
Killip class III, n (%)	2 (2.1)
Symptoms-reperfusion time (min)*	181 [135–181]
**Angiographic data**	
Anterior infarction, n (%)	39 (41.1)
Initial TIMI 0/1 flow, n (%)	76 (80.0)
Collateral grades 2/3, n (%)	20 (21.0)
Multivessel disease, n (%)	31 (32.6)
Thrombectomy, n (%)	44 (46.3)
Final TIMI 3 flow, n (%)	87 (91.6)
Final TIMI 2 or 3 flow, n (%)	94 (98.9)
**Medical treatment**	
IIB-IIIA GP inhibitors, n (%)	55 (57.9)
β-Blocker at discharge, n (%)	88 (92.6)
ACE inhibitors at discharge, n (%)	86 (90.5)
ACE inhibitors follow-up, n(%)	85 (89.5)
Statins at discharge, n (%)	94(98.9)

* Data expressed in minutes as median [25^th^–75^th^ percentiles].

ACE: Angiotensin Converting Enzyme.

**Table 3:** sACE2 laboratory results.

Group	Baseline sACE2	7 days sACE2
Controls	74.9 [62.8–87.5]	–
STEMI patients	104.4 [87.4–134.8]*	115.5 [92.9–168.6]**

Data expressed as relative fluorometric units per microliter per hour (RFU/µl/hr).

* p<0.001 for comparison between baseline values in controls and STEMI patients by Mann-Whitney U test.

** p<0.01 for comparison between baseline and 7 days values in patients by paired Wilcoxon test.

sACE2: Serum Angiotensin Converting Enzyme 2.

**Figure 3:**
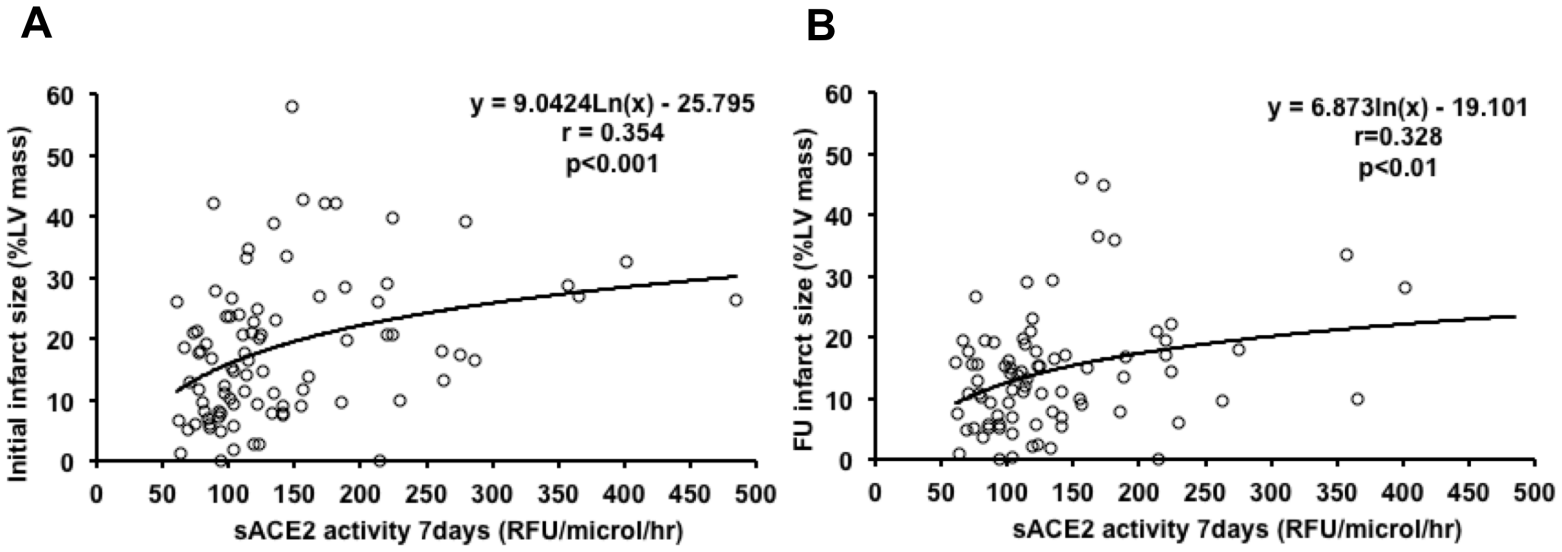
Correlation between sACE2 activity and infarct size. A weak but significant correlation was observed between infarct size and sACE2 activity at 7 days (panel A) and at 6 months follow-up (panel B). FU: Follow-up. sACE2: Serum Angiotensin Converting Enzyme 2; LV: left ventricular; RFU: relative fluorometric units.

We found sACE2 activity at 7 days to be increased in 19 patients in Killip class 2 or 3 during admission as compared to patients in Killip 1 (181.5 [136.4–224.1] vs 104.5 [86.0–137.6] RFU/µl/hr, respectively, p<0.001. A correlation was found between sACE2 activity at 7 days and the myocardium at risk derived from T2 W imaging, (r = 0.373, p<0.001). The results were similar when only the angiographic measurements of myocardium at risk where taken into account for all patients. Infarct size derived from ce-CMR weakly correlated with sACE2 activity at 7 days (r = 0.354, p<0.001) and 6 months follow-up (r = 0.328, p<0.01), (Figure 3). sACE2 activity at 7 days was not significantly different between patients with and without MO on CMR (121.0 [92.2–174.9] vs 114.6 [91.7–159.5] RFU/µl/hr respectively, p = 0.556. We found no correlation between sACE2 activity at 7 days and MO score (r = 0.159, p = 0.129).

Serum ADAM17 was not significantly different between controls and patients at baseline (1001 [477–1646] vs 1067 [822–1536] pg/ml, p = 0.437). ADAM17 was not correlated with sACE2 activity at baseline (r = −0.103, p = 0.351) or 7 days (r = 0.041, p = 0.712). We found no association between serum ADAM17 and infarct size by ce-CMR at 7 days (r = 0.047, p = 0.666) or 6 months (r = −0.015, p = 0.898).

### Predictors of Late Systolic Function

**Figure 4:**
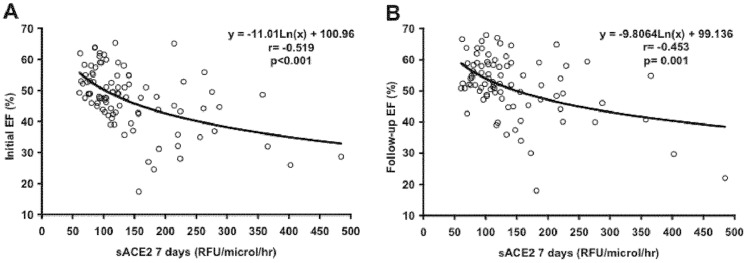
sACE2 activity and LV systolic function. Scatter-plots showing a statistically significant correlation between ACE2 activity at 7 days and EF during admission (panel A) and at 6 months follow-up (panel B). sACE2: Serum Angiotensin Converting Enzyme 2; EF: ejection fraction; RFU: relative fluorometric units.

Infarct size by ce-CMR (r = −0.705, p<0.001) or peak troponins (r = −0.675, p<0.001), MO score (r = −0.493, p<0.001), the initial BNP (r = −0.404, p<0.001), sACE2 at 7 days (r = −0.350, p<0.01) and the myocardium at risk (r = −0.304, p<0.01) were univariate predictors of late EF. Figure 4 shows the correlation between sACE2 activity at 7 days and acute or follow-up EF. By multivariate linear regression analysis, ACE2 activity at seven days and infarct size by ce-CMR were the only two independent predictors of late EF (Table 4).

**Table 4:** Predictors of follow-up EF by multivariate analysis.

r^2^ model	Variable	β coefficient	95% CIof β	t	p value
r^2^ = 0.643	Infarct size (%LV)	−0.470	−0.691:−0.248	−4.240	**<0.001**
	sACE2 activity(7 days)	−0.025	−0.048:−0.002	−2.218	**0.030**
	BNP	−0.007	−0.018∶0.05	−1.162	0.250
	Myocardiumat risk	0.059	−0.097∶0.216	0.76	0.450
	MO extent	−0.364	−1.548∶0.819	−0.615	0.541
	Troponin peak	−0.005	−0.018∶0.008	−0.740	0.462
	Sex	−4.532	−9.316∶0.252	−1.894	0.063

BNP: B-type natriuretic peptide; MO: microvascular obstruction; sACE2: Soluble Angiotensin Converting Enzyme 2; CI: confidence interval.

### ACE2 Activity and Remodeling

Although many variables including myocardium at risk, infarct size, MO extent, ACE2 activity and BNP correlated with LV ESDi and EDVi at baseline and follow-up, ACE2 activity was the only predictor of % increase in EDVi (r = 0.291, p<0.01). Fifteen (16.9%) subjects presented adverse LV remodeling at follow-up, defined as an increase ≥20% in the follow-up EDVi as compared to baseline. They had increased ACE2 activity at 7 days (156.6 [118.2–231.1] vs 111.7 [86.5–142.6] RFU/µl/hr, p = 0.011). However, infarct size and MO extent were not significantly different between both groups (18.4±12.8% vs 17.2±11.0% and 1.7±2.3 vs 1.2±1.7 segments respectively, p = ns for both). Neither BNP nor peak troponins were different in both groups (see Table S1 for further details). Furthermore, differences in LV remodeling can be illustrated when subdividing the 88 subjects with follow-up CMR into tertiles based on the sACE2 activity found at 7 days. As shown in Table 5, the proportion of patients with heart failure during admission or adverse LV remodeling increased according to sACE2 activity tertiles. A significant linear association was observed between EF and infarct size among sACE2 activity tertiles. In general, mean EF improved on follow-up among the three groups. However, different adaptive changes in ESVi and EDVi were observed according to sACE2 tertiles. The odds ratio of predicting adverse LV remodeling with sACE2 activity above the third tertile after adjustment for age was 4.4 (1.3 to 15.2, p = 0.027 by Fisher’s Exact Test).

**Table 5:** Clinical characteristics based on tertile plasma sACE2 activity at 7 days.

Variable*	sACE2 tertile 1	sACE2 tertile 2	sACE2 tertile 3	P value†
sACE2 (RFU/µl/hr)	<100.7	100.7–140.8	>140.8	–
Age (yrs)	59.8 [55.4–64.2]	56.4 [51.7–61.1]	58.3 [53.8–62.8]	0.560
Killip>1 (%)	3.1	17.9	41.9	**0.001**
Multivessel dis. (%)	18.8	46.4	36.7	0.068
Diabetes	15.6	17.9	29.0	0.380
Hypertension	43.8	35.7	35.5	0.747
ACE inhibitors (%)	90.6	92.9	87.1	0.755
β-Blockers (%)	84.4	92.9	100.0	0.066
CMR characteristics				
Infarct size (%LV)	13.4 [10.0–16.7]	16.9 [13.2–20.6]	23.9 [18.9–28.9]	**0.001**
Initial EF (%)	53.4 [51.2–55–6]	48.5 [45.8–51.2]	40.7 [36.7–44.7]	**<0.001**
Follow-up EF (%)	55.9 [53.5–58.3]	53.4 [50.5–56.3]	46.6 [42.3–51.0]	**<0.001**
% Δ EDVi	1.6 [−4.5–7.8]	4.1 [−4.6–12.8]	14.7 [6.2–23.1]	**0.043**
% Δ ESVi	−3.6 [−9.2–1.9]	−4.7 [−15.1–5.8]	6.9 [−4.3–18.3]	0.147
LV remodeling (%)	6.9	10.7	33.3	**0.017**
MO score	0.8 [0.4–1.3]	1.5 [0.8–2.2]	2.0 [1.1–2.8]	0.057

* Continuous variables expressed as mean [95% confidence interval].

† Analysis of variance across sACE2 quartiles, chi-square test for nominal variables. CMR = cardiac magnetic resonance; EF = ejection fraction; EVDi = end-diastolic volume index; ESVi = end-systolic volume index; LV = left ventricular; MO = microvascular obstruction.

LV remodeling is defined as an increase in EDVi≥20%.

## Discussion

There are several novel findings in this study. First, ACE2 is upregulated in the acute phase of STEMI in humans and can be determined non-invasively by measuring sACE2 activity. Second, sACE2 activity was found to correlate with the infarct size. Finally, in addition to infarct size, ACE2 activity measured acutely is associated with late LV systolic function and occurrence of adverse LV remodeling.

A significant activation of the RAAS occurs after myocardial infarction, leading to a marked increase in Angiotensin-II generation by ACE, which promotes fibrosis and marked adverse LV remodeling [16]. However, despite optimal medical treatment with ACE inhibitors or Angiotensin-II receptor blockers, many patients develop adverse remodeling and heart failure. It follows that additional medical treatment seems desirable. In the last decade, significant attention has been paid to the role of ACE2 in cardiac disease and hypertension. In the heart, ACE2 is the primary pathway for the metabolism of Angiotensin-II by cleavage into Angiotensin-(1–7), a peptide with opposed biological actions as compared to ACE [17]. The genetic loss of ACE2 in mice causes increased levels of Angiotensin-II and progressive mild LV dilatation with concomitant systolic dysfunction that is restored by ACE deletion, suggesting that this response is mediated by an increase in Angiotensin-II levels.

The role of ACE2 in acute myocardial infarction has been addressed in experimental studies. There is evidence that ACE2 protein is increased in the infarct-related and peri-infarct myocardium [8], [18]. Interestingly, treatment with ramipril had no effect on cardiac ACE2 mRNA expression or ACE2 activity, which remained elevated. ACE2-deficient mice showed greater infarct size, contractile dysfunction and more severe LV dilatation that persisted 8 weeks after myocardial infarction [18]. On the other hand, overexpression of cardiac ACE2 achieved by lentivirus transfection prior to myocardial infarction conferred early ischemic protection, including a significantly decrease in infarct size assessed by ce-CMR and marked recovery of contractility in the infarct and the remote non-infarcted myocardium [19]. Taking these studies together, it seems reasonable to summarize that ACE2 loss post-infarction is detrimental and ACE2 overexpression confers cardioprotection against adverse LV remodeling and contractile dysfunction.

In humans, there is evidence for an upregulation of ACE2 gene expression and activity in failing hearts with ischemic and non-ischemic cardiomyopathy, and among diabetic patients with heart disease, suggesting a regulatory role of the enzyme in advanced heart failure [9], [20]. An increase in Angiotensin-(1–7) forming activity was found in the heart of subjects with end-stage idiopathic cardiomyopathy that was abolished with the specific ACE2 inhibitor C16. In addition, the presence of ACE2 protein was confirmed by Western blot analysis in those hearts [21]. Serum sACE2 activity is measured as the result of a proteolytic shedding effect in cell membranes, giving the opportunity to track this pathway activation non-invasively. Using this methodology, Epelman et al. showed increased sACE2 activity among patients with a clinical diagnostic of heart failure [7]. Moreover, sACE2 activity correlated with the NYHA class, LV ejection fraction and B-type natriuretic peptide levels. This relationship was observed among patients with ischemic and non-ischemic cardiomyopathy at different stages, again suggesting a compensatory mechanism to limit LV dysfunction. In line with these studies, sACE2 activity correlated with the severity of LV dysfunction in other conditions, such as Chagas’ disease. Remarkably, plasma ACE2 activity was equally potent as BNP to predict cardiac death and heart transplantation [22]. In our study, the enzyme activity is related to infarct size and particularly with the ischemic territory at risk, which parallels the association between ACE2 loss and stunning/hibernation described in experimental models. Although ACE2 is mainly localized in the endothelium, we found no association between sACE2 and the presence or severity of microvascular obstruction on ce-CMR, as it would have been expected in cases of severe endothelial damage. One plausible explanation for this finding might be the impaired tissue perfusion observed in areas of severe microvascular obstruction, which limits the enzyme leakage into the blood stream. ACE2 activity in plasma has been shown to increase in the first week in a murine model of non-reperfused myocardial infarction [23]. Our study is the first to describe a proportional upregulation of sACE2 activity to infarct size and LV dysfunction in human myocardial infarction. ACE and ACE2 activation following STEMI may influence fibrosis deposition, finally leading to adverse LV remodeling. In this study, the upregulation of ACE2 seen during the acute phase was also correlated with fibrosis on ce-CMR measured at 6 months follow-up. However, more precise methods to depict interstitial diffuse fibrosis by ce-CMR as a result of RASS system activation were not applied in our study. ACE2 activity was significantly higher among patients that developed marked dilatation (ΔEDVi >20%) between the baseline and follow-up ce-CMR study. Importantly, no differences were found in infarct size between patients who developed or not marked dilatation during the study. Therefore, a subset of patients with small infarct size and near normal EDVi at baseline with optimal medical treatment may be at risk for developing adverse remodeling and could be identified based on non-invasive determination of sACE2 activity. Regardless of this marginal clinical utility, the ultimate goal of measuring sACE2 activity depends on the ability to target medically the activation of ACE2-Angiotensin (1–7) pathway, rather than serving as a non-invasive tool to predict the occurrence of adverse LV remodeling following myocardial infarction.

There are several limitations in this study. The sACE2 activity was higher in patients than controls at baseline. However, enzyme activity was not repeated in controls at 7 days. Herein, the possibility of sACE2 increase observed among patients being a random finding cannot be totally ruled out. Whether the increase in circulating ACE2 is the cause or the effect of LV remodeling following STEMI remains uncertain. Cleavage of membrane ACE2 by up-regulated proteases in advanced heart failure, such as tumor necrosis factor-∝ ADAM17 may deplete local myocardial ACE2 causing LV remodeling. We found no differences in serum ADAM17 between patients and controls. In addition, ADAM17 did not correlate with sACE2. However, we did not measure ADAM17 and ACE2 in the myocardium, and therefore a local shedding effect causing increased sACE2 cannot be excluded. Alternatively, release of membrane ACE2 in plasma may represent a compensatory mechanism to limit heart failure by means of increasing systemic Angiotensin (1–7) against Angiotensin-II actions. Measurements of circulating Angiotensin-II or Angiotensin 1–7 were not available and therefore, our study do not provide the mechanism for the supposed protective role of ACE2 in STEMI, that will need further evaluation. We performed the initial CMR a mean of 6 days after the index event, when most of LV remodeling has probably occurred. In addition, we did not include clinically unstable patients in whom the ce-CMR study could not be performed. Those are the patients with more adverse remodeling and larger infarct size. These facts might have limited the true relationship found between infarct size, LV remodeling and sACE2 activity. As a result, the correlations found between sACE2, infarct size, LV EF or remodeling were modest. However, our purpose was to demonstrate a relationship between ACE2 activation and LV dysfunction to support future therapeutic developments rather than using the measure as a clinical tool to predict adverse remodeling.

## Conclusions

To our knowledge, this is the first study reporting that sACE2 activity is increased in the acute phase of STEMI in humans and correlates with the extent of jeopardized myocardium and infarct size. ACE2 activity measured acutely seems to be an independent predictor of late LV systolic function and it is also associated with the occurrence of adverse remodeling. Further pathophysiological and mechanistic studies are warranted to elucidate whether this association, that has been shown to be present in several cardiac conditions causing left ventricular dysfunction, may become a new target for therapy following STEMI.

## Supporting Information

Table S1 **CMR characteristics of patients with adverse LV remodeling on follow-up as compared to the remaining.**
(DOC)
