# Supplementary material for: Role of Circulating Angiotensin Converting Enzyme 2 in Left Ventricular Remodeling following Myocardial Infarction: A Prospective Controlled Study
Source: PLoS One. 2013 Apr 22;8(4):e61695. doi: 10.1371/journal.pone.0061695 (PMC3632515; doi:10.1371/journal.pone.0061695)
Supplement: Table S1 — CMR characteristics of patients with adverse LV remodeling on follow-up as compared to the remaining. (DOC) [file pone.0061695.s001.doc]

**Table S1. CMR characteristics of patients with adverse LV remodeling on follow-up as compared to the remaining.**

|  | **Δ EDVi ≥20% absent (n=73)** | **Δ EDVi ≥20% present (n=15)** |
| --- | --- | --- |
| sACE2 7 days (RFU/µl/hr) | 111.7 [86.5-142.6] | 156.6 [118.2-231.1]** |
| BNP (pg/ml) | 115.2 [51.5-204.1] | 87.0 [45.9-178.4] |
| Troponins peak | 107.2 [41.9-178.4] | 70.2 [30.5-257.8] |
| Infarct size (% by CMR) | 16.3 [8.6-23.7] | 16.5 [7.8-26.9] |
| MO score | 1.2 ± 1.7 | 1.7 ± 2.3 |
| EDVi baseline | 80.9 [70.2-94.2] | 71.8 [57.1-88.0]* |
| ESVi baseline | 38.6 [33.4-48.0] | 36.2 [26.3-46.1] |
| EDVi 6 months | 79.0 [70.2-92.9] | 99.5 [85.0-116.8]** |
| ESVi 6 months | 36.4 [29.2-45.5] | 52.7 [39.1-63.7]** |
| EF baseline (%) | 48.9 [43.7-55.0] | 47.8 [42.5-51.0] |
| EF 6 months (%) | 53.4 [49.6-58.9] | 45.4 [40.0-55.0]* |

Data expressed as median [25^th^-75^th^] percentiles, except MO score, expressed as mean±standard deviation.

* P<0.05 and ** P<0.01 for comparison with U Mann-Whitney test.
